# Topical aluminum chloride as a treatment option for Hailey-Hailey disease: a remarkable therapeutic outcome case report^⋆^

**DOI:** 10.1016/j.abd.2023.03.009

**Published:** 2023-11-24

**Authors:** Maraya de Jesus Semblano Bittencourt, Pedro Carneiro Marinho, Thereza Christina Frade, Gabriela Athayde Amin, Lorena Silva de Carvalho, Lívia Eloi Castro Santos

**Affiliations:** Department of Dermatology, Centro Universitário do Estado do Pará, Belém, PA, Brazil

*Dear Editor,*

Hailey-Hailey disease (HHD) or benign familial pemphigus, is a rare autosomal dominant genodermatosis, characterized by chronic and painful blisters and ulcerations in intertriginous areas, which cause significant impairment of quality of life of affected patients.^1^, ^2^ This case report describes a 55-year-old woman who had developed painful, recurrent lesions since the age of 13, which started as blisters in the intertriginous areas. Over the years, the patient remained undiagnosed and underwent treatments with oral and topical corticosteroids, topical and systemic antifungals, oral and topical antibiotics, and topical immunomodulators, with slight improvement and frequent relapses, which resulted in significant impairment of her quality of life.

On dermatological examination, multiple ulcerous-crusted plaques were observed, some with intact blisters, disseminated in intertriginous areas of the neck, dorsum, abdomen, groin and axillae (Figs. 1A, 2A and 3A). Histopathological examination showed a “dilapidated brick wall” appearance of the epidermis, compatible with HHD (Fig. 4). The patient was advised to use, exclusively on the lesion areas, 15% aluminum chloride in aqueous solution twice a week (Perspirex® roll-on), with almost complete remission after eight weeks, as well as substantial improvement in quality of life (Figs. 1B, 2B and 3B). The patient is being monitored, with isolated and minor recurrences.

**Figure 1:**
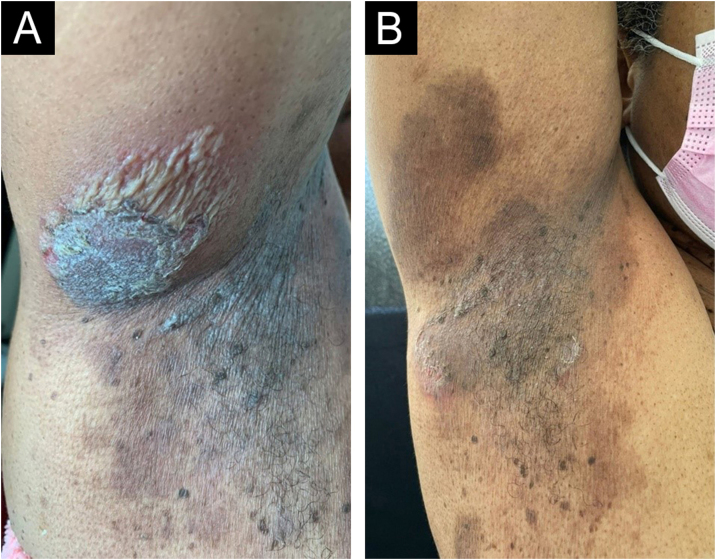
Right axilla before (A) and after (B) eight weeks of treatment. Considerable reduction in ulcerous-crusted plaques and blisters, with a predominance of residual hyperchromia at the end of treatment.

**Figure 2:**
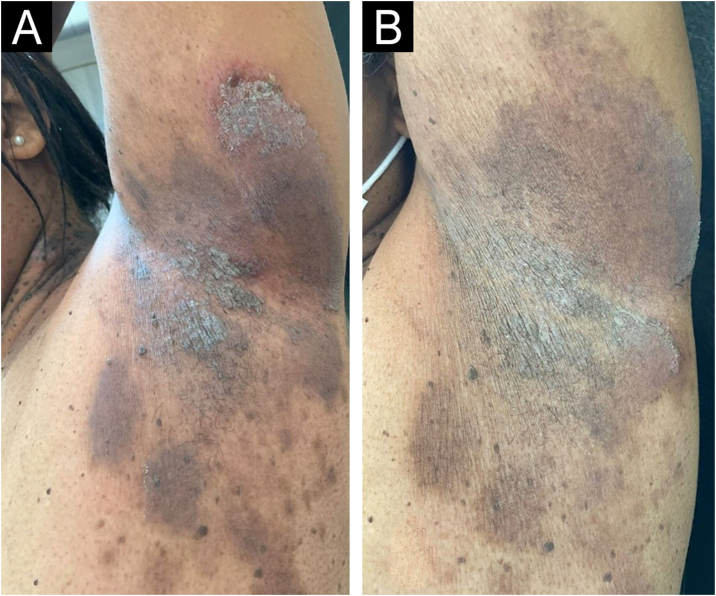
Left axilla before (A) and after (B) eight weeks of treatment. Decrease in ulcerous-crusted plaques and blisters, and predominance of residual hyperchromia at the end of treatment.

**Figure 3:**
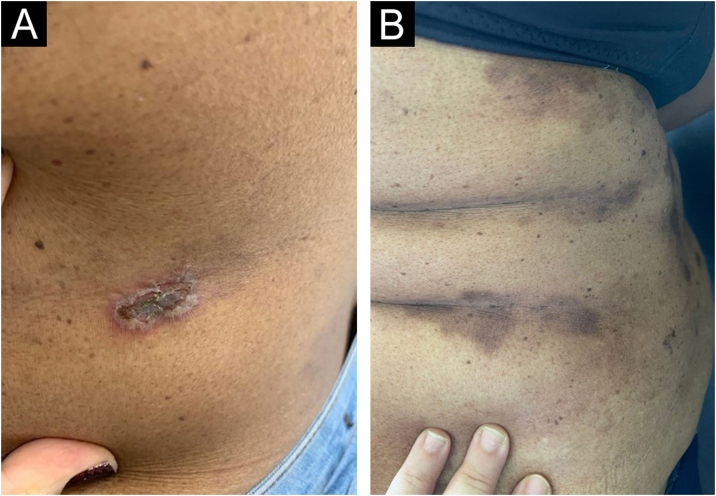
Abdomen before (A) and after (B) eight weeks of treatment. Considerable reduction in ulcerous-crusted plaques and blisters, and predominance of residual hyperchromia at the end of treatment.

**Figure 4:**
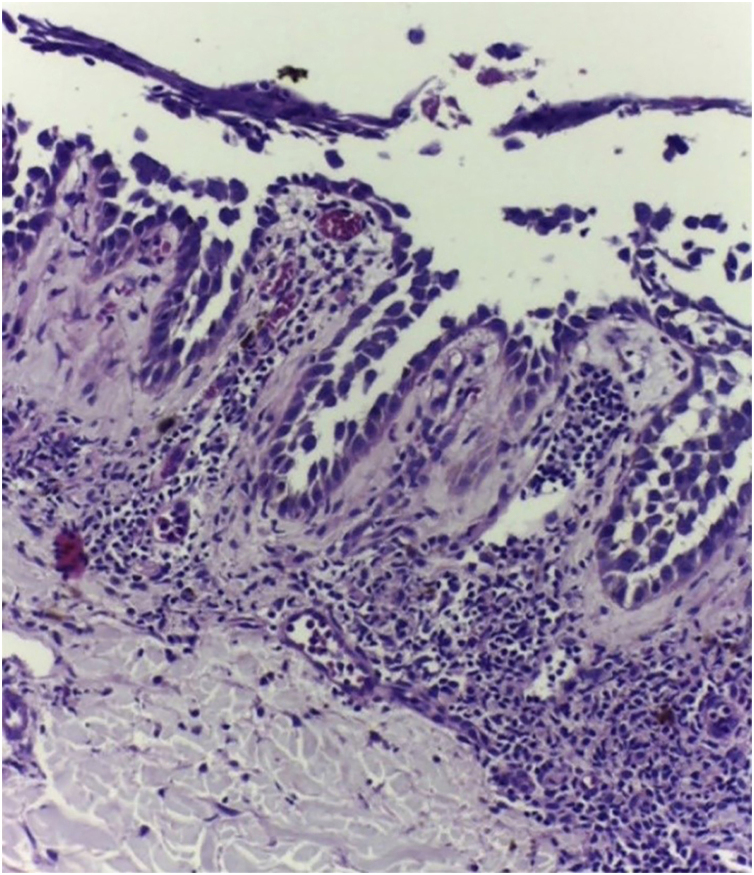
Light Microscopy with extensive acantholysis with a “dilapidated brick wall" appearance of the epidermis (Hematoxylin & eosin, ×400).

HHD lesions usually appear soon after puberty, compromising patients active life years.^3^, ^4^ Patients quality of life is significantly impaired due to the symptoms (pruritus, burning sensation and pain), foul body odor and chronicity.^2^, ^4^ Mutations in the ATP2C1 gene, which encodes a Ca^2+^ ATPase pump, lead to changes in Ca^2+^-dependent intracellular signaling, resulting in loss of cell adhesion in the epidermis.^1^

Several topical and systemic treatments are proposed in the literature,^3^ such as laser ablation, botulinum toxin type A,^4^, ^5^ dermabrasion, narrow-band UVB phototherapy, topical 5-fluorouracil, dupilumab,^6^ TNF-alpha inhibitors, as well as oral treatment with oxybutynin, apremilast, vitamin D, glycopyrrolate, afamelanotide, dapsone, acitretin and cyclosporine.^1^, ^3^ Some of these treatment options are high-cost, others have important side effects and others are not very accessible for most patients.

Sweating is known to worsen and may precipitate the appearance of blisters in patients with HHD, which is why treatments that block sweat production, such as botulinum toxin,^4^, ^5^ oral oxybutynin, and oral glycopyrrolate have been used with good response in some cases.^3^ For this reason, treatment with a topical 15% aqueous aluminum chloride solution was chosen. This medication acts at the level of the eccrine gland duct, blocking it and producing atrophy and vacuolation of the secretory glandular cells. An “ionic” effect of the used treatment is also possible, by providing another element (aluminum) in contact with the skin, where intracellular traffic of Ca^2+^ and Mg^2+^ ions is altered by the defect caused by the disease.^7^

The patient in this report showed an excellent clinical response, with no local side effects and a considerable reduction in the frequency and intensity of crises. The authors consider this treatment option to be low-cost and effective in reducing the frequency of crises and symptoms of this disabling condition.

## Financial support

None declared.

## Authors' contributions

Maraya de Jesus Semblano Bittencourt: Collection, analysis and interpretation of data; drafting and editing of the manuscript; effective participation in research orientation; intellectual participation in the propaedeutic conduct of the studied case; approval of the final version of the manuscript.

Pedro Carneiro Marinho: Collection, analysis and interpretation of data; critical review of the literature; drafting and editing of the manuscript; intellectual participation in the propaedeutic conduct of the studied case.

Thereza Christina Frade: Approval of the final version of the manuscript; critical review of the literature; drafting and editing of the manuscript.

Gabriela Athayde Amin: Approval of the final version of the manuscript; critical review of the literature; drafting and editing of the manuscript.

Lorena Silva de Carvalho: Approval of the final version of the manuscript; critical review of the literature; drafting and editing of the manuscript.

Lívia Eloi Castro Santos: Critical review of the literature; intellectual participation in the propaedeutic conduct of the studied case.

## Conflicts of interest

None declared.

## References

[1] Rogner D.F., Lammer J., Zink A., Hamm H. (2021). Darier and Hailey-Hailey disease: update 2021. J Dtsch Dermatol Ges.

[2] Silveira K.S., Zac R.I., Oliveira P.J., Neves D.R., Barbosa V.G., Café M.E. (2009). Caso para diagnóstico [Hailey-Hailey disease. Case for diagnosis]. An Bras Dermatol.

[3] Ben Lagha I., Ashack K., Khachemoune A. (2020). Hailey-Hailey disease: an update review with a focus on treatment data. Am J Clin Dermatol.

[4] Bessa G.R., Grazziotin T.C., Manzoni A.P., Weber M.B., Bonamigo R.R. (2010). Hailey-Hailey disease treatment with Botulinum toxin type A. An Bras Dermatol.

[5] Kothapalli A., Caccetta T. (2019). Botulinum toxin type A for the first-line treatment of Hailey-Hailey disease. Australas J Dermatol.

[6] Alzahrani N., Grossman-Kranseler J., Swali R., Fiumara K., Zancanaro P., Tyring S. (2021). Hailey-Hailey disease treated with dupilumab: case series. Br J Dermatol.

[7] Micaroni M., Giacchetti G., Plebani R., Xiao G.G., Federici L. (2016). ATP2C1 gene mutations in Hailey-Hailey disease and possible roles of SPCA1 isoforms in membrane trafficking. Cell Death Dis.

